# Substituting Maize with Blends of *Solanum tuberosum* Chips and *Ensete ventricosum* Corm on Egg Production and Quality in Bovans Brown

**DOI:** 10.1002/vms3.70464

**Published:** 2025-07-28

**Authors:** Yisehak Kechero, Gossaye Weji, Taju Hussein

**Affiliations:** ^1^ Department of Animal Sciences College of Agricultural Sciences Arba Minch University Arba Minch Ethiopia; ^2^ Department of Animal and Range Sciences College of Agriculture Wolaita Sodo University Sodo Ethiopia

**Keywords:** egg quality, enset corm, hen‐day egg production, layer, potato chips, substitute

## Abstract

The conventional cereal grains that human beings compete for, along with proteins that are exceedingly costly in tropical conditions, make up the majority of the poultry rations. Productivity may increase by using non‐conventional feed resources since they lower production costs. The experiment was conducted in the Sodo district of the Gurage zone, in the central highlands of Ethiopia, to ascertain the effects of substituting enset (*Ensete ventricosum*) corm and potato (*Solanum tuberosum*) chips for corn on the quantity (58.75 ± 0.81) and quality of eggs laid by Bovans Brown layers. A total of 132 Bovans brown layers (1.67 ± 0.033 kg, Mean ± SD) aged 24 weeks were used in the investigation. In place of maize grain, the layers were fed feed that contained 0% (T1), 15% (T2), 30% (T3) and 45% (T4) of an enset corm and potato chip mixture at a 2:1 ratio. The trial was totally randomized and comprised four treatments. With 11 birds per replicate, each treatment was carried out three times. A 12‐week experiment was conducted. Hens were weighed at the start and end of the trial. Dry matter intake, hen‐day egg production (HDEP), egg weight and egg mass were all tracked daily. Every 15 days, four eggs per replicate were evaluated for four different egg quality metrics (eggshell weight and thickness, albumen weight and height, Haugh unit and yolk weight and colour). A mix of potato chips and enset corms with 3568 kcal/kg DM, 4.28% CP, 0.17% ether extract and 2.40% crude fibre. The average daily weight gains of T1 (2.08 g), T4 (1.99 g) and T3 (1.85 g) were comparable and significantly larger (*p* < 0.05) than T3 (1.28 g). The feed conversion ratios among treatments were statistically different (*p* < 0.05). The HDEP of T1 (81.08%) was significantly lower (*p* < 0.05) than that of other treatments that included varying inclusion rates of potato chips and enset corms. Compared to T1 and T3, T4 showed a higher eggshell weight (*p* < 0.05). The findings of this study suggest that enset corm and potato chips can be used in layer (Bovans Brown) rations to partially replace maize; further research is necessary to replace maize entirely (≥50%–100%) with enset corm and potato chips.

## Graphical abstract

The average daily weight gains of T1 (2.08 g), T4 (1.99 g) and T3 (1.85 g) were comparable and significantly larger (*p* < 0.05) than T3 (1.28 g). Statistically different egg weight, egg shape index, egg albumen weight, albumen height, egg shell weight, egg yolk color, feed conversion ratio and hen day egg production.

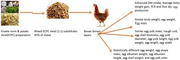

## 1 Introduction

Most impoverished countries in the tropics rely heavily on scavenging methods for poultry production. Because of the use of this management style, there has been a high death rate and low productivity. While village chicken production systems in tropical locations face major challenges, they play a vital role in rural communities' livelihoods. Due to a variety of technological issues, the productivity of poultry farming in developing nations per unit of bird and the contribution of this sector to the national economy of the country is relatively low. Feed shortages and the high cost of conventional energy and protein sources are the primary problems limiting poultry output in Ethiopia (Atawodi et al. 2008; Abera et al. 2011). Feed materials utilized in commercial poultry production are high‐quality grains that are also consumed by humans. This puts the sector in direct competition for grains, raising the cost of poultry production (Gura 2008; Mengesha 2011).

Ethiopia has unique agro‐climatic conditions that promote the cultivation of many different types of food and feed crops, resulting in a diversified range of alternative feeds ideal for poultry feeding (Tadelle et al. 2002). *Ensete ventricosum* is one such crop that can potentially be supplemented in poultry feed. The most significant components of the enset are the pseudo‐stem, corm and stalk of the inflorescence (Adugna 2008). More than 70% of the enset plant's parts are pseudo‐stem and corm (Ajebu et al. 2008). The corm, or underground stem, of enset, is employed for vegetative propagation of the plant. It is a sustainable food supply that may be uprooted and consumed at any moment during the plant's life cycle, especially during a prolonged drought. Enset corm contains a lot of highly soluble carbohydrates and starch but not much fibre or cellulose (Mohammed et al. 2013). The average enset dry matter, taken as a whole plant, contains 9.13% ash and 90.87% organic matter (Mohammed et al. 2013). Again, there is 60.62% starch, 7.4.57% soluble carbohydrates, 0.84% crude fat, 9.48% crude fibre and 5.98% crude protein (CP). The crop's higher energy yield (238.9 kcal/kg DM) is another well‐known characteristic (Pijls et al. 1995). Although records indicate that it can produce up to 12 t ha^−1^ year^−1^, it normally produces 4 to 7 t ha^−1^ year‐1 (Hiebsch 1996). Ajebu et al. (2008) reported that one of the best options for chicken feeding might be enset green due to its year‐round availability. Driessen et al. (2001) also reported thigh biomass output (5200 kg ha^−1^, or 33 kg plant1) at a density of 1600 plants ha^−1^.

Potato (*Solanum tuberosum*) has a great production capacity (fresh tuber yield, 20 tons/ha), whereas maize and wheat yield 5 and 7 tons/ha, respectively, and can adapt to many soil types. It is an energy source that could replace maize or other cereals used for layer feeding. The energy value of potato meal used is more than 2830 ME per kg. On a dry matter basis, potato meal comprises more than 65% starch, 6.74% ash, 12.72% CP, 23.48% CF, and 0.99% EE. Potato meal is said to be less expensive than maize and wheat (PAN 1995). As a result, looking for locally available non‐traditional feed resources such as enset and potato to design poultry rations may be necessary.

Regardless of the method of production, the cost, availability and quality of feed ingredients significantly restrict the capacity to produce animals for the general public through chicken farming. Feed cost is sometimes blamed for the high price of chicken products; it is believed to account for more than half of the entire cost of intensive chicken production (Wilson and Beyer 2000). Maize grain has been employed as an energy source in poultry diets, raising the cost of poultry feed. The increasing demand for energy and protein for human consumption has sparked research interest, particularly in developing countries, in the quest for locally accessible alternative feed ingredients for chicken feeding that are relatively cheaper. To enhance chicken productivity, poultry producers must look beyond cereal grains (Chauynarong et al. 2009; Swain et al. 2014). In order to evaluate if the partial substitution of maize with the mixtures of enset corm and potato chips in the diets of laying Bovans brown chickens at rates of 0%, 15%, 30% and 45% would affect egg quality and quantity while improving the feed conversion ratio, the current study was designed to find out the effects of partly replacing maize with a combination of enset corm and potato chips on feed intake, body weight changes and feed conversion ratio in Bovans brown layers, as well as the effects of doing so on egg production and egg quality traits in Bovans brown layers. This research project has been approved by the institutional research compliance committees for animal ethics and welfare, in addition to the biosafety and biosecurity committees that review and authorize research‐related activities.

The hypothesis of this research was that the MECPC used to substitute the main cereal of the diet 9maize) could affect hen production and the effect could depend on the type of dietary treatments used.

## 2 Materials and Methods

### 2.1 About the Study Area

The experiment was carried out in the Sodo district of Gurage Zone, in the central Ethiopian highlands. It can be found at 8°18′5′ N and 38°32′48′ E, around 105 km south of Addis Ababa. The research area's mean annual temperatures and rainfall are 17°C and 1021.7 mm, respectively. The elevation range is 1700–3460 meters above sea level. The farming activity includes the production of crops and the care of livestock. In the study area's highlands, enset and potatoes are the principal crops grown for domestic use (SWSNRMO 2018).

### 2.2 Experimental Feed Preparation and Treatments

Farmers in Sodo district, Sewati Kebele, Central Ethiopia, sold us enset plants of the “Ashakti” kind that were 5–6 years old. After cutting off the aerial portion of the enset plant, a fresh enset corm was scooped out. Whole fresh enset corms were washed, cleaned and sifted before being peeled, cut into little slices and sun‐dried for five days over a plastic sheet. In order to avoid uneven drying and degradation, it was constantly turned. Potatoes were purchased from a neighbourhood market. Skins were removed after thorough filth and dust cleaning. A potato chunk was then exposed to sunlight to dry. The right precautions were taken to avoid the growth of fungus when drying the potatoes. Following mechanical grinding of the sun‐dried potato and the acquisition of feed components from a nearby market, the following substances were used to create the study's rations: maize grain, enset corm and potato chips mixed at a 2:1 ratio, wheat middling, noug seed cake, soybean meal, vitamin premix, salt, limestone and dicalcium phosphate. The following items were ground to pass through a 5 mm sieve: dried enset corm, dried potato, maize grain, nougat seed cake, salt and limestone.

The chemical compositions of the major feed constituents were identified from representative samples. The four treatment rations used in the current experiment were developed to be nearly iso‐caloric and iso‐nitrogenous, with 2800 kcal ME/kg DM and 16.0% CP, in order to meet the nutrient needs of layers (Leeson and Summers 2008). In Ration 1 (control), neither test feed, potato chips, nor enset corm was employed. The amounts of enset corm and potato chips in the rations for treatments 1, 2, 3 and 4 were 0% enset corm and potato chips 15% (10% enset corm and 5% potato chips), 30% (20% enset corm and 10% potato chips) and 45% (30% enset corm and 15% potato chips), respectively (Table 1). Soybean meal, nougat seed cake, wheat middling, limestone, vitamin premix, dicalcium phosphate and salt were all ingredients in the treatment meals. Water is accessible at all times in individual troughs for each pen.

**TABLE 1:** Proportion of the feed ingredients used in formulating layers rations.

Ingredients	Treatments, means			
	T1	T2	T3	T4
Maize	43.5	37	30.5	19.5
ECPCM	—	6.5	13	24
Wheat middling	18.5	17.5	16.5	15.5
Noug cake	15	16	16	16.5
Soybean meal	14	14	15	15.5
Vitamin premix*	1	1	1	1
Salt	0.5	0.5	0.5	0.5
Lime stone	7	7	7	7
Dicalcium phosphate	0.5	0.5	0.5	0.5
Total	100	100	100	100

*Note*: T1, 0% MECPC; T2, 15% MECPC; T3, 30% MECPC; T4, 45% MECPC as substitute to maize.

### 2.3 Management of Experimental Birds

With the aid of wire mesh and 10 cm‐thickened sawdust, 12 pens, each measuring 2×1.4 meters, were created inside the experimental home. The experimental pens, watering apparatus, feeding troughs and laying nests were properly cleaned, disinfected and treated against external parasites before the actual experiment got underway. The chickens received vaccinations against common poultry diseases such as fowl pox and Lasota. Along with water; multivitamin tablets and oxytetracycline (20 g/100 L of water) were administered. All through the experiment, free access to clean water was provided. For the experiment, 132 Bovan Brown chicken layers were employed, each weighing 1.67 ± 0.033 kg (Mean ± SD) and having a lifespan of 24 weeks. Golden Poultry Production and Multiplication Centre provided the meat for the study in the form of pulled chicken. To give the birds time to adjust, they were kept in houses for seven days. Each treatment group of chickens was fed a different treatment diet during this time. Four treatments were given a total of 33 layers each in a completely randomized design (CRD); each treatment was repeated three times and included 11 layers with a stocking density (Solomon et al. 2018) of 8 birds per m^2^.

### 2.4 Laboratory Analysis

In order to pass past a 1 mm screen for chemical analysis, dried feed samples were milled. Following the proximate technique of analysis, samples were examined for dry matter, CP, ether extract, crude fibre and ash (AOAC 2019). This analysis was performed in the animal nutrition lab at Jimma University. In the chemistry lab at Arba Minch University, the contents of calcium and total phosphorus were measured using the atomic absorption and vanado‐molybdate methods, respectively (AOAC 2019). By subtracting 100 from (CP + CF + EE + ash), the nitrogen‐free extract (NFE) was calculated. By using the Kjeldal technique to quantify nitrogen, CP will be computed by multiplying N by 6.25. The experimental diets' metabolizable energy (ME) content was determined using an indirect method based on the following Wiseman (1987) equation: ME (Kcal/kg DM) = 3951 + 54.4EE − 88.7CF − 40.8 ash.

#### 2.4.1 Feed Intake

Every day, at the same times of 6:00 and 15:00, a weighted amount of feed was provided, and fresh tap water was always available. The feed was provided in hanging circular plastic feeders that were around the height of the birds' backs. A circular plastic drinker containing water was also available. In accordance with the recommendations of the manufacturer, vitamins were administered through drinking water. There was a separate nest in each pen, which was coated with sawdust. After removing external impurities by ocular inspection, orts were collected and weighed. The feed offered and refused for each replication were noted and multiplied by the appropriate DM content. The difference between the feed offered and rejected on a per‐DM basis was used to calculate the amount of feed consumed. Samples of feed were provided and rejected daily based on treatments; these samples were stored for three months and then bulked up for chemical analysis.

#### 2.4.2 Body Weight Change and Feed Conversion Ratio

Body weights (BW) were recorded both at the beginning and conclusion of the trial. The difference between the final and starting BW was used to calculate the body weight change. To calculate the total amount of feed consumed, the amount of leftover feed was removed and deducted from the amount fed after feeding time. Giving the animal a predetermined amount of feed based on its 2.5% body weight allowed for this to be achieved. The weight of feed consumed per egg mass was calculated to obtain the feed conversion ratio for each replicate. According to Melkamu (2017), the average feed conversion ratio for each treatment was calculated as the average of the replicates for each treatment.

#### 2.4.3 Egg Production

Three times a day, at 7:00 a.m., 12:00 a.m. and 6:00 p.m., eggs were removed from each pen. At the conclusion of the time period, the three collections' combined total as well as the number of birds active each day were noted and compiled. Using the average results from each duplicate and applying Hunton's technique (1995) and Rahayu and Widjastuti (2019), the rate of lay for each treatment was calculated as the average percentage of hen‐day egg production as follows:

$$\begin{array}{ccc} & & \%\;\mathrm{Hen}-\mathrm{day}\;\mathrm{egg}\;\mathrm{production}\\ & & \;=\frac{\mathrm{Number}\;\mathrm{of}\;\mathrm{eggs}\;\mathrm{collected}\;\mathrm{per}\;\mathrm{day}\;\times \;100}{\mathrm{Number}\;\mathrm{of}\;\mathrm{hens}\;\mathrm{presented}\;\mathrm{that}\;\mathrm{day}} \end{array}$$

#### 2.4.4 Egg Weight and Egg Mass

By dividing the total egg mass by the number of eggs, the average egg weight was computed. For each treatment, daily collected eggs were weighed by sensitive balance immediately after collection.

#### 2.4.5 Egg Quality Parameters

The egg quality indicators, such as egg size, egg width, egg weight and eggshell thickness, as well as albumen and yolk height and weight, were taken at intervals of two weeks. The size, colour and height of four freshly deposited eggs per replicate were measured. A total of 192 eggs—48 for each treatment—were used for the quality examination. A 0.0‐gram‐sensitive digital scale was used to measure the weight of the eggs. The egg's length and width were measured using a digital caliper. The following equation was used to estimate the Shape Index (SI): Shape Index = [egg width/egg length] × 100 (Anderson et al. 2004; Duman et al. 2016). The internal measurements were collected after the egg sizes were measured externally by carefully poking a hole through the egg's pointed end large enough to allow the albumen and yolk to pass through without mingling their contents. Next, the albumen was carefully separated from the yolk, which was then put in a petri dish for weighing. The corresponding albumen was simultaneously placed on a different petri dish and weighed. The weight difference between the Petri plates used to weigh the egg contents after and before the egg component was used to calculate the weight of the egg components.

Before the subsequent weigh‐in, the Petri dishes were cleaned in clean water after each weigh‐in and dried. Digital calipers were used to measure the height of the albumen and yolk. By carefully inserting the opened section into the shell and weighing it on a digital balance, the weight of the shell and membrane was determined. Three different locations (the large end, the small end and the equator region of the eggshell) was used to measure the thickness (mm) of the shell with intact membranes, and the average value was acquired using a digital vernier caliper. The following equation was used to determine the yolk index (Elnagar and Abd‐Elhady 2009):

$$\mathrm{Yolk}\;\mathrm{diameter}\;\left(\mathrm{cm}\right)=\left[\mathrm{Yolk}\mathrm{height}\left(\mathrm{cm}\right)/\mathrm{Yolk}\mathrm{index}\left(\%\right)\right].$$

The colour of a correctly mixed yolk sample placed on white paper was compared to 1–15 Roche fan measuring colour strips, which vary from pale to orange‐yellow in colour, to determine the yolk's colour. Using the Haugh (1937) formula, the Haugh unit was determined from the egg weight and albumen height.

Albumen quality was quantified by the Haugh unit (HU) (Keener et al. 2006). The shattered eggs' albumen and yolk were meticulously sorted. The height of the albumen and yolk was measured using a tripod micrometre. The weight of Albumen was determined using a digital balance. The following formula was used to determine the HU (Raji et al. 2009).

$$\mathrm{HU}=100\;\left(H+7.5\;1.7W0.37\right)$$

where, HU stands for the Haugh unit, H for albumen height, and W for egg weight (in grams).

### 2.5 Statistical Analysis

The statistical analysis software system (SAS Institute Inc. 2008 version 9.2) used the general linear model (GLM) method to do an analysis of variance (ANOVA) on the obtained data. The least significant difference (LSD) was used to differentiate the meaningful difference between the treatment means. At *p* < 0.05, the treatment effect was deemed significant. The Kolmogorov–Smirnov test was used to test the null hypothesis that the data follows a normal distribution. The experiment made use of the following model:

$$Y_{\mathrm{ij}}=\mu +\alpha_{i}+\sum_{\mathrm{ij}}$$

where, Y_ij_ = represents the j^th^ observation in the i^th^ treatment level, *μ* = overall mean, *α*_i_ = treatment effect, ∑_ij_ = random error.

## 3 Results

### 3.1 Chemical Makeup of Ingredients in Feed and Experimental Rations

The chemical composition and ME value of feed ingredients and treatment meals, respectively, are shown in Tables 2 and 3. When compared to other feed items, the mixture of enset corm and potato chips had the lowest CP (4.28%), EE (0.17%) and NFE value (88.73%). The soybean had the highest CP value, followed by the nougat seedcake. Maize had the highest ME value, followed by a blend of enset corm and potato chips and wheat middling, while nougat cake had the lowest value. The treatment diets had similar amounts of CP. The ME values of the treatment diets were comparable, while T4 was somewhat lower.

**TABLE 2:** The chemical composition of feed stuffs (on dry matter basis).

	Chemical composition of feed stuffs (%)				
Ingredients	MECPC	Maize	NSc	Wheat middling	SBM
DM (%)	92.19	90.29	93.62	90.93	96.32
CP (%DM)	4.28	8.16	28.76	12.64	45.50
EE (%DM)	0.17	3.80	11.91	1.96	4.95
Ash (%DM)	4.42	3.18	10.18	3.62	5.92
CF (%DM)	2.40	3.70	20.12	7.80	12.30
Ca (%DM)	0.04	0.04	0.04	0.20	0.02
P (%DM)	0.38	0.72	0.71	0.51	0.36
ME (kcal/kg DM)	3568	3695	1930	2924	2887
NFE	88.73	81.19	29.03	73.98	31.33

Abbreviations: Ca, calcium; CF, crude fiber; CP, crude protein; DM, dry matter; EE, ether extract; ME, metabolizable energy; MECPC, mixture of enset corm and potato chips; NFE, nitrogen free extract; NSC, Noug seed cake; P, phosphorus.

**TABLE 3:** Chemical composition (% DM, unless specified) of experimental diets.

	Treatments, Means			
Nutrients	T1	T2	T3	T4
Dry matter (%)	92.52	92.67	92.84	93.01
Crude protein	16.57	16.48	16.56	16.55
Crude fiber	7.79	7.83	7.79	7.79
Ether extract	4.50	4.36	4.15	3.98
Ash	16.25	16.42	16.44	16.54
Calcium	3.22	3.22	3.22	3.22
Phosphorus	0.65	0.63	0.61	0.59
Nitrogen free extract	54.89	54.88	55	55.14
ME (kcal/kg DM)	2841.95	2823.75	2815.13	2801.71

*Note*: T1 = 0% MECPC; T2 = 15% MECPC; T3 = 30% MECPC; T4 = 45% MECPC as substitute to maize.

### 3.2 Feed Intake and Egg Laying Performance

The dry matter intake of treatments 2 and 3 was similar (*p* > 0.05) while treatments 1 and 4 were significantly higher and lower (*p* < 0.05) when compared with T2 and T3, respectively (Table 4). The mean body weight gain was found to be higher in T4 compared to other treatments (*p* < 0.05), while treatments 1 through 3 showed no significant difference (*p* > 0.05). The T3 and T4 diets produced more hen‐day eggs (*p* < 0.05) than the T1 diet, while no significant difference was observed between T2 and T3 (*p* > 0.05). Egg mass and weight were comparable (*p* > 0.05) among treatments. Layers given T1 (control) and T2 had a significantly higher (*p* < 0.05) feed conversion ratio than T3 and T4.

**TABLE 4:** Dry matter intake, body weight change and egg laying performance of Bovans brown hens fed mixture of enset corm and potato chips as substitute to maize.

	Treatment, mean					
Variables	T1	T2	T3	T4	SEM	*p*‐value
DM intake (g/hen/d)	119.62^a^	118.38^b^	118.86^b^	116.03^c^	0.142	0.040
Initial body weight(kg)	1.67	1.68	1.65	1.67	0.001	0.891
Final body weight(kg)	1.86^a^	1.85^ab^	1.77^b^	1.85^ab^	0.002	0.100
Body weight gain (g/hen/day)	2.08	1.85	1.28	1.99	0.273	0.302
Egg weight(g)	58.05	58.35	58.47	58.32	0.834	0.950
Hen day egg production (%)	81.08	83.34	84.07	83.61	2.553	0.180
Egg mass (g/hen/day)	49.61	49.04	49.53	49.52	0.221	0.120
FCR (g feed DM/g egg)	2.47^a^	2.42^a^	2.40^b^	2.40^b^	0.001	0.026

Abbreviatons: FCE, feed conversion efficiency; SEM, standard error of the mean.

^a,b^ Means within a row with different superscripts differ (*p* < 0.05); T1, 0% ECPCM; T2, 15% ECPCM; T3, 30% ECPCM; T4, 45% ECPCM as a substitute to maize.

### 3.3 Egg Quality Parameters

Except for egg weight, egg shape index, egg albumen weight, egg albumen height, eggshell weight and egg yolk colour, all other egg quality parameters were similar (*p* > 0.05) for layers fed rations containing 0%, 15%, 30% and 45% enset corm and potato chip mixture diets. Eggshell weight was in the order T4> T1>T3> T2 (*p* < 0.05).

## 4 Discussion

### 4.1 Chemical Composition of Feedstuffs

Although MECPC had marginally lower ME than maize, blending it with potato chips at 2:1 could have sufficient ME to promote the layer's performance. Also, the ME energy in the MECPC was found to be greater than other feed items commonly included in poultry feed, like wheat middling, soybean meal and nougat seedcake. MECPC, on the other hand, has lower crude fibre and crude protein levels than the other feed constituents. The CP content of the enset corm and potato chips mixture (4.28%) in the current study was greater than the CP values of enset corm (2.2%) published by N. Ajebu and Eik (2014) and kocho (3.8%), reported by Nigussu et al. (2019). Melese (2013) reported a higher CP (4.76%) content of kocho than the current trial. The discrepancy in CP content could be attributed to differences in the enset types used to manufacture kocho. N. Ajebu et al. (2008) discovered a variation in the CP content of unprocessed corm and pseudostem from which kocho is formed. In general, the CP content of enset corm is low, indicating the necessity for supplementation with CP‐rich feed sources such as nougat cake and soybean meal. On the other hand, the ME content of the enset corm and potato chips combo was higher than that of the other feed items employed in this experiment, with the exception of maize grain, which has a comparable value.

The ME content of the enset corm and potato chips obtained in this study was higher than that found by Mohammed et al. (2013), who discovered that unprocessed corm and pseudostem have 3437 and 3379 kcal/kg DM, respectively. However, it was equal to the 3647 kcal/kg DM reported for kocho by Nigussu et al. (2019). Its potential to replace the energy value of cereals like maize in layer diets is justified by the ME value of the enset corm and potato chip mixture determined in this study. The treatment meals' calcium and phosphorus concentrations were determined to be within the suggested ranges for layering. The combined enset corm and potato chip CF content (2.4%) was comparable to the values (2.3%) reported by Nigussu et al. (2019) for kocho and low compared to the unprocessed corm (5.65%) and pseudostem (7.51%) reported by Mohammed et al. (2013) for different enset varieties but within the range of 1.47%–4.42% reported by Melese (2013) for different enset varieties.

The relatively low amount of CF in the mixture of enset corm and potato chips and the high level of ME indicates that it has the potential to be used as a dairy ingredient in layer rations. According to NRC (1994), the energy and protein requirements of laying hens range from 2700 to 2850 kcal/kg dry matter and from 14.0% to 19.0%, respectively. The CP concentrations of the treatment meals ranged from 16.48% to 16.57%, which was greater than the NRC's (1994) recommended minimum CP requirement of 14%. Also, the ME content of the treatment meals ranged from 2802 to 2842 kcal/kg DM, which was higher than the NRC's (1994) minimum ME requirement of 2700 kcal/kg DM for the white leghorn layer.

### 4.2 Feed Intake and Laying Performance

There is no published data on feed intake, egg production or egg quality in layers fed a ration including a combination of enset corm and potato chips. In the current study, Bovans brown layers fed 45% maize (T1) without enset corm and potato chips had DM intake comparable to or equal to that of rations containing a 45% blend of enset corm and potato chips (T4). The capacity of mixtures of ECPC to replace maize in terms of intake and digestibility may account for the similarities between a treatment consisting of 45% ECMC (T4) and a treatment consisting of 45% maize (T1). A. Ajebu and Abebash (2015) reported similar dry matter intake for Hubbard grill chickens fed 0%, 33%, 67% and 100% kocho diets, as did Seyoum (2013) for those fed 0%, 33.3%, 66.7% and 100% furfurame, a by‐product of kocho processing produced from enset, as a replacement for maize. Sultana et al. (2016) revealed that including potato meal in the layers diet resulted in slightly higher egg production and a better feed conversion ratio in the control group, which could be attributed to the hens' increased feed consumption compared to the potato meal‐fed hens. Again, this lower egg production could be attributed to the high fibre content of potato meal, which results from birds' poor nutrient utilization.

The lack of considerable variation in hen‐day egg production suggests that a diet including up to 45% enset corm and potato chips as a replacement for maize could be employed to meet the nutrient needs of layers. Enyenihi et al. (2009) found that replacing maize with 75% cassava tuber meal increased HDEP. Inclusion of sweet potato meal up to 20% as a replacement for maize enhanced HDEP in white leghorn layers compared to 30% and 40% diets, which could be attributed to the effect of anti‐nutritional elements at higher levels of potato diets (Ravindran 1995). According to Saentaweesuk et al. (2000), the entire substitution of maize for cassava in layers ration had no effect on output performance. Ladokun et al. (2007) found that layers fed 50% sweet potato meal rations had greater HDEP than those fed solely maize. Afolayan et al. (2013) demonstrated that substituting sweet potato meal for maize had no effect on the health of the layers. Nigussu et al. (2019) found that strata that ingested a higher level of kocho had higher HDEP. Kana et al. (2013) discovered no influence on egg weight or feed conversion in pullets fed 0%, 33%, 66% or 100% cassava root meal in place of maize. Aderemi et al. (2012) discovered that birds fed diets with more than 25% cassava meal had worse feed conversion. The average daily increase, Hen‐day egg output, egg weight and egg mass of layers fed varied levels of enset corm and potato chips mixed diet were equivalent (*p* > 0.05). The lack of substantial variation in hen‐day egg production in the current study suggests that up to 45% MECPC diet as a replacement for maize might be employed to fulfil the nutrient requirements of layers.

### 4.3 Egg Quality

As shown in Table 5, the egg quality parameters for egg weight, egg shape index, egg albumen weight, egg albumen height, eggshell weight and egg yolk colour were significant for Bovans brown hens fed enset corm and potato chips, with substitution rates for maize at 0%, 15%, 30% and 45%. Other parameters for egg quality were similar across treatments. This indicates that without affecting the parameters governing egg quality, combinations of potato chips and enset corm can be used in place of maize in the number of layers. According to Aderemi et al. (2012), the effects of substituting whole cassava meal for maize on internal and external egg quality metrics ranged from similar to significant. Furthermore, all treatment groups showed similar effects (*p* > 0.05) from the partial substitution of enset corm for maize on the egg quality of white leghorn layer indicators (Nigussu et al. 2016).

**TABLE 5:** Egg quality characteristics of Bovans brown hens fed mixture of enset corm and potato chips.

	Treatments, mean					
Egg quality parameters	T1	T2	T3	T4	SEM	*p*‐value
Sample egg weight (g)	56.74^b^	57.17^ab^	58.08^ab^	58.75^a^	0.81	0.042
Egg length (mm)	54.37	54.72	54.79	54.78	0.106	0.392
Egg width (mm)	43.04	42.82	43.03	43.48	0.183	0.354
Egg shape Index	79.16^a^	78.26^b^	78.52^b^	79.36^a^	0.526	0.027
Egg yolk weight (g)	16.00	15.88	15.98	16.36	0.104	0.348
Egg albumin weight (g)	34.53^b^	34.83^b^	35.65^ab^	36.17^a^	0.435	0.050
Egg albumin height (mm)	7.57^b^	8.03^a^	7.98^ab^	8.06^a^	0.050	0.032
Egg yolk height (mm)	17.92^b^	18.15^ab^	17.96^b^	18.36^a^	0.450	0.119
Egg yolk diameter (mm)	40.12	39.39	39.68	40.07	1.490	0.867
Egg shell thickness (mm)	0.35	0.35	0.35	0.34	5.833	0.344
Egg shell weight (g)	5.94^b^	6.11^ab^	6.04^b^	6.27^a^	0.008	0.011
Egg yolk color (RSP)	2.44^a^	2.23^ab^	2.02^b^	2.52^a^	0.038	0.041
Haugh unit	87.49	89.94	89.40	89.67	1.692	0.166
Egg yolk index	0.45	0.46	0.45	0.46	0.006	0.836

Abbreviations: SEM = Standard Error of the Mean; *RSP = Roche scale points;.

^a,b^ Means within a row with different superscripts differ (*p* < 0.05);T1 = 0% ECPCM; T2 = 15% ECPCM; T3 = 30% ECPCM; T4 = 45% ECPCM as a substitute to maize.

## 5 Conclusion

The dietary inclusion of the mixes of enset corm and potato chips (MECPC) in the layer ration had an effect on dry matter intake (ranging from 118.4–121.8 g/hen/d), feed conversion ratio (2.47 in T1 to 2.40 in T4 feed DM/g egg) or hen‐day egg production (61.1 in T1 through 64.2% in T4) (SEM = 0.018). Likewise, with the inclusion of MECPC in the diets except for egg weight, egg shape index, egg albumen weight, egg albumen height, eggshell weight and egg yolk colour, all other egg quality parameters were similar. This indicates the capacity for substituting maize with MECPC in layers. Therefore, a 45% inclusion of MECPC in the diet of Bovan layers partially affected the production or quality of the eggs.

## Author Contributions

**Yisehak Kechero Kechero**: conceptualization, data curation, software, supervision, writing – review and editing. **Gossaye Weji**: funding acquisition, writing – original draft, writing – review and editing. **Taju Hussein**: funding acquisition, writing – original draft, writing – review and editing.

## Ethics Statement

The authors affirm that the journal's ethical rules, as stated on the author guidelines page, were followed. However, the study did not use animals, but rather a questionnaire survey, field observation, and measurement. As a result, data collection was carried out following a verbal agreement with the agricultural and locality administration offices, as well as the research's target farmers.

## Conflicts of Interest

The authors declare no conflicts of interest.

## Peer Review

The peer review history for this article is available at https://www.webofscience.com/api/gateway/wos/peer‐review/10.1002/vms3.70464.

## Data Availability

The datasets created and analysed during the current investigation are available upon reasonable request from the corresponding author.
